# *Amblyomma cajennense* is an intrastadial biological vector of *Theileria equi*

**DOI:** 10.1186/1756-3305-6-306

**Published:** 2013-10-23

**Authors:** Glen A Scoles, Massaro W Ueti

**Affiliations:** 1 USDA, ARS, Animal Disease Research Unit, 3003 ADBF, Washington State University, Pullman, WA 99164, USA

**Keywords:** Equine piroplasmosis, Tick-borne transmission, Vector competence, *Dermacentor variabilis*, *Amblyomma cajennense*, *Theileria equi*, Intrastadial transmission

## Abstract

**Background:**

The apicomplexan hemoprotozoan parasite *Theileria equi* is one of the etiologic agents causing equine piroplasmosis, a disease of equines that is endemic throughout large parts of the world. Before 2009 the United States had been considered to be free of this parasite. Occasional cases had occurred but there was no evidence for endemic vector-borne transmission in the U.S. until a 2009 outbreak in Texas in which *Dermacentor variabilis* and *Amblyomma cajennense* were implicated as vectors. Although *D. variabilis* has previously been shown to be a competent laboratory vector, studies suggested *A. cajennense* was not a competent transstadial vector, even though the presence of this tick species on horses in South American is epidemiologicaly correlated with higher a prevalence of infection. In this study we tested the transstadial and intrastadial vector competence of *D. variabilis* and *A. cajennense* for *T. equi*.

**Methods:**

A tick passaged *T. equi* strain from the Texas outbreak and ticks colonized from engorged females collected off horses on the outbreak ranch in Texas were used for these studies. Nymph or adult ticks were fed on infected horses and transmission fed on naïve horses. Infections were tracked with PCR and serology, dissected tick tissues were tested with PCR.

**Results:**

*A. cajennense* transmitted *T. equi* intrastadially when adult ticks acquired infection by feeding on an infected horse, and transmitted to a naïve host on subsequent reattachment and feeding. *D. variabilis* failed to transmit in the same experiment. Transstadial transmission was not successful for either tick species. PCR on DNA isolated from eggs of females that had fed on an infected horse suggests that there is no transovarial passage of this parasite by either tick species.

**Conclusion:**

This work confirms that ticks from the Texas population of *A. cajennense* are competent intrastadial vectors of *T. equi*. We propose that the most likely natural mode of transmission for this parasite/vector combination in the Texas outbreak would have been biological transmission resulting from adult male ticks moving between infected and uninfected horses. The intrastadial mode of transmission should be considered as one equally possible scenario whenever implicating vectors of *T. equi*.

## Background

*Theileria equi*, also known by some authors as *Babesia equi* and originally described in 1901 by Laveran as *Piroplasma equi*, is one of the etiologic agents causing equine piroplasmosis (EP). *T. equi* infects wild and domestic equines worldwide, with the exception of a few countries including Australia, Canada, Great Britain, Ireland, Japan and New Zealand, which are considered to be free of infection [1]. To prevent the spread of EP these and several other countries restrict international and/or internal movement of horses based on serological testing.

Ticks are obligate hosts for sexual stage development of *T. equi* and, as is usually the case for biological transmission, the vector relationship is restricted to a few tick species that are competent to support this portion of the lifecycle of the parasite. To complete its life cycle the parasite undergoes a complex series of developmental events in its tick vector. This developmental cycle is similar to that of other apicomplexan hemoprotozoan parasites. Steps in this process include: acquisition of haploid merozoites during blood-feeding on an infected host; formation of sexual stages in the gut and fusion of these gametes to form a diploid zygote; transformation of the zygote into a motile kinete, which moves to, and invades, the salivary glands where a reduction division results in the formation of haploid sporozoites; and finally, replication of sporozoites in the salivary glands and subsequent transmission in the saliva as the tick feeds [2,3]. This series of events is responsible for biological transmission of *T. equi* and is usually thought of as occurring transstadially with the immature tick acquiring the parasite and the subsequent tick stage transmitting. However, we know that this can also occur intrastadially in adult male *Rhipicephalus (Boophilus) microplus*, resulting in intrastadial biological transmission [4].

Upon initial infection following tick transmission, susceptible equine hosts develop acute disease characterized by anorexia, fever and anemia. Following resolution of acute disease animals retain a persistent asymptomatic infection for life. Parasitemia during persistent infection is 10^4^-10^6^ copies per ml [5], well below levels that are routinely detectable on stained blood smears. Consequently, these asymptomatic carriers are detected primarily by serology. Because persistently infected horses can serve as reservoirs for tick-borne transmission [5], transportation of these asymptomatic horses can lead to the introduction of *T. equi* into regions that are free of EP. However, endemic tick-borne transmission can occur only in regions where competent biological vectors are present. While mechanical transmission can and does occur [6], tick-borne biological transmission is important because it increases the probability of transmitting from persistently infected horses with very low parasitemia by creating an opportunity for amplification of parasites in the salivary glands of the tick vector.

The U.S. horse industry has a direct economic impact on the U.S. economy that is valued at $39 billion per year (http://www.horsecouncil.org/national-economic-impact-us-horse-industry), and EP is of significant importance to the industry, because it is a barrier to free international movement of horses, both for international sporting events and for trade. A large outbreak of EP was identified in the United States in 2009 [7]; prior to this the U.S. had been considered by The World Organization for Animal Health (Office International des Epizooties, OIE) to be free of endemic EP since eradication of the disease here in 1988 (http://www.oie.int/wahis/public.php?page=home). The EP free status of the U.S. was due, at least in part, to the fact that there was no evidence for endemic vector-borne transmission. Some of the sporadic cases that had been identified in the U.S. prior to the 2009 outbreak may have been the result of diagnostic insensitivity at importation because the complement fixation test [8], used for import screening prior to 2005, has a high incidence of false negative results [9]. Prior to the Texas outbreak vector-borne transmission had not been suspected for any of the U.S. cases. In fact, only 2 tick species found in the United States, *Dermacentor variabilis* and *R. microplus*, had previously been shown to be competent experimental vectors of *T. equi*[10,11]. *Amblyomma cajennense* has been suspected as a vector throughout endemic areas of South America based on epidemiological evidence [12-14], however, laboratory transmission by this species had never been demonstrated prior to the U.S. outbreak [7].

In the current study we report work to confirm that the ticks from the Texas population of *A. cajennense* are competent intrastadial biological vectors of *T. equi*, but we have been unable to transmit this parasite transstadially with this tick species. On the other hand, although *D. variabilis* has been shown to be a competent intrastadial vector in previous studies, we have been unable to transmit with this tick species in the current study, suggesting that the vector capacity of *D. variabilis* is low. In this study we have begun to define the efficiency and mode of *T. equi* transmission by these two tick species.

## Methods

### Ticks

Colonies of *D. variabilis* and *A. cajennense* were established from ticks collected off horses in 2009 at the outbreak ranch in Kleeburg Co., Texas; some ticks were fully engorged, others were partially fed and were allowed to reattach and feed to repletion on horses [7]. Eggs laid by 14 engorged female *D. variabilis*, and 24 engorged female *A. cajennense* were used to start each respective colony. For each species, eggs from individual females were mixed and divided into weighed aliquots in glass vials with screen tops for hatching. Many of the ticks of each species had been collected from horses that were seropositive for *T. equi*, therefore it was necessary to confirm that the tick colonies were free of *T. equi* infection. Although this parasite is not known to be transovarially transmitted by *R. microplus*[4] data is lacking for other tick species. Consequently, F_1_ larval offspring were fed on naïve horses and the horses monitored for infection by PCR and serology to confirm that there was no transmission (data not shown), subsequently these tick colonies were maintained on cattle. All of the experiments described in this paper were conducted with nymphal and adult ticks reared from these F_1_ larval offspring.

Tick feeding was accomplished as previously described by placing the ticks inside a stockinet sleeve or cloth feeding patch attached with cattle hip tag cement to the backs and/or sides of their hosts [11]. Before and after feeding, ticks were held in humidified chambers (98% RH) in an incubator at 26˚C, with a light cycle of 12 hours light: 12 hours dark.

### Animals

All horses used in these experiments were cared for following protocols approved by the University of Idaho Institutional Animal Care and Use Committee (Protocol #2010-54). Horses were purpose bred Shetland-cross ponies 1–2 years of age with no prior exposure to ticks or to *Babesia* or *Theileria* infections. Prior to use, horses were confirmed to be sero-negative for *T. equi* using the commercially available cELISA (VMRD Inc., Pullman, WA). Unless otherwise noted all horses were spleen intact. As noted below, a single splenectomized horse was used in one experiment. After transmission feedings horses were monitored for infection with daily temperature and packed cell volume (PCV), blood samples were taken for PCR and serology 3 times per week. Horses that did not seroconvert and/or become PCR positive within 75 days of exposure were considered to be free of infection.

### Parasite isolate

All of these experiments were conducted with an isolate of *T. equi* that originated via tick transmission from the outbreak ranch in Kleeburg Co, Texas. Horse Ho-183 acquired infection with this parasite isolate when fed on by *D. variabilis* that had been collected from seropositive horses on the outbreak ranch, which confirms that this isolate of *T. equi* is tick transmissible [7]. Microsatellite typing showed that Ho-183 was infected with a single clone of *T. equi* (TE-0035) [15], and all of the subsequent infections of horses used for acquisition feeding in these experiments were the result of needle passage of 100 ml of blood from this horse. Frozen blood stabilates of this *T. equi* isolate were made from Ho-183 during acute infection and are available for future studies and for archival purposes.

### Detection of infection

For each experiment described below, a sample of ticks was dissected and their guts and/or salivary glands were tested by PCR for infection with *T. equi*. Methods for dissection and DNA isolation from blood and dissected tick tissues have been previously described [5]. Both the nested PCR (nPCR) and the quantitative PCR (qPCR) assays used in this work target the single copy EMA-1 gene of *T. equi* and have also been described previously [5,16]; nPCR results represent the number of *T. equi* genome copies calculated to be present in each ml of blood. Since these published assays were designed from sequences of the Florida lab strain of *T. equi* the EMA-1 target region of the Texas strain used in these studies was cloned and sequenced to confirm that there was no variation at the primer and probe sites. A 816 base fragment of the EMA-1 gene [17] was cloned and sequenced from four different blood samples from horses infected with this Texas *T. equi* strain: 1) Ho-183 during acute infected (immediately after the initial tick transmission); 2) Ho-183 after one year of persistent infection; 3) from a horse infected by needle passage from Ho-183 and; 4) from a horse infected by tick passage from Ho-183; all EMA-1 sequences had >99.5% identity with one another (only 4 single base changes in a 816 base sequence) and with the prototype sequence in GenBank (accession number: L13784); most importantly, there was no sequence variation at the primer or probe sites.

### Experimental design

Two different transmission scenarios were tested on both acutely and persistently infected horses: i. Transstadial Transmission - nymphal acquisition-adult transmission (also known as interstadial transmission); and ii. Intrastadial Transmission - adult acquisition-adult transmission by males and/or partially fed females moved between hosts and allowed to reattach and continue feeding. The potential for transovarial passage - nymphal or adult female acquisition followed by transovarial passage to the eggs, was tested by assay for the presence of parasite DNA in eggs of exposed females.

### Transstadial transmission, experiment 1

Nymphal *A. cajennense* and *D. variabilis* were acquisition fed to repletion on two different *T. equi* infected horses, Ho-183 (persistently infected) and Ho-198 (in the acute phase of infection, infected by needle passage from Ho183) (Table 1); both tick species were fed on each horse. Replete nymphs of each species were held until they molted to the adult stage. Approximately 14 days after molting *A. cajennense* adults from the two different acquisition hosts were pooled and then divided between two spleen intact horses, Ho-259 and Ho-261. Each horse received both male and female ticks in separate feeding patches for a 7-day transmission feed; numbers of ticks applied can be found in Table 2. Approximately 100 days after molting to adults, *D. variabilis* males and females from each of the acquisition horses were all transmission fed on a single horse (Ho-138) for 7 days (Table 3). After transmission feeding salivary glands were dissected from a sample of ticks from each group and tested by PCR for the presence of *T. equi.*

**Table 1:** **Transstadial transmission Experiment 1, nymphal acquisition feeding of *A. cajennense *** **and *D. variabilis *** **on *T. equi *** **infected horses**

**Acquisition host**	**qPCR**^**a **^**(copies/ml)**	**Species**	**Number of nymphs fed**	**Molted adults**	
				**♂♂**	**♀♀**
Ho-183	1.4 × 10^4^	*D. variabilis*	53	34	17
		*A. cajennense*	189	NA^b^	NA^b^
Ho-198	2.1 × 10^5^	*D. variabilis*	33	17	16
		*A. cajennense*	198	211^c^	160^c^

^a^At the time of nymphal repletion (5/26/10).

^b^Pooled with ticks from Ho-198, see note “c” below.

^c^Total of the *A. cajennense* pooled from both acquisition hosts, Ho-183 and Ho-198.

**Table 2:** **Transstadial transmission, Experiment 1, *A. cajennense *** **adults held 14 days after molting from nymphs fed on *T. equi *** **infected horses shown in Table **1

**Transmission horse ID**	**Ticks recovered**^**a**^		**Ticks dissected & tested**^**b**^	
	**♀♀**	**♂♂**	**♀♀**	**♂♂**
Ho-259	40/41	50/53	0/40	0/50
Ho-261	37/41	43/53	0/37	0/43

Transmission fed for seven days on two different transmission horses. Numbers of ticks applied, recovered and dissected from each horse after transmission feeding. There was no transmission.

^a^Number recovered/number applied.

^b^Number positive over the number tested.

**Table 3:** **Transstadial transmission, Experiment 1, *D. variabilis *** **adults held 100 days after molting from nymphs fed on *T. equi *** **infected horses shown in Table **1

**Acquisition horse**	**Ticks recovered**^**a**^		**Ticks dissected & tested**^**b**^	
	**♀♀**	**♂♂**	**♀♀**	**♂♂**
Ho-183	16/17	32/34	0/15	0/32^c^
Ho-198	16/16	17/17	0/15	0/16

Transmission fed for seven days on two different transmission horses. Numbers of ticks applied, recovered and dissected from each horse after transmission feeding. There was no transmission.

^a^Number recovered/number applied.

^b^Number positive over the number tested.

^c^The first 18 ticks dissected at the same time as the others, the remaining 14 dissected ≈ 3 weeks later.

### Transstadial transmission, experiment 2

A second transstadial transmission experiment was performed for *A. cajennense* to allow a longer incubation period between molt and adult feeding and a longer transmission feeding time. Nymphs were acquisition fed to repletion on horse Ho-266 during the acute phase of infection and held for molting as described for the first trial above. Approximately 50 days after molting to adult, males and females were transmission fed in separate feeding patches for 14 days on Ho-212. In this experiment ticks were sampled and tested by PCR at multiple time points after acquisition feeding, including as fed nymphs, as freshly molted adults and before and after transmission feeding; adult ticks were dissected and both guts and salivary glands were tested by PCR, nymphs were not dissected (Table 4).

**Table 4:** **Transstadial transmission experiment 2, *A. cajennense *** **nymphs fed to repletion on *T. equi *** **infected horse Ho-266**

**Sex**	**Fresh adult gut**	**35 day adults**		**Transmission fed (# recovered/# applied)**	**Tested after transmission**	
		**Gut**	**SG**		**Gut**	**SG**
♂♂	0/10	0/20	0/20	9/90	0/9	0/9
♀♀	0/10	0/21	0/21	96/97	0/15	0/15

Parasitemia (as estimated by qPCR) during time of nymphal feeding 1.16 × 10^5^ genome copies per ml. Ticks were sampled at various intervals before and after transmission feeding (freshly molted adults, adults after 35 days, and adults after transmission feeding). 50 days after molting ticks were applied to Ho-212 for transmission feeding.

### Intrastadial transmission, experiment 1

*A. cajennense* and *D. variabilis* males and females were fed on a splenectomized horse, Ho-226, that had been infected with *T. equi* 6 days prior to application. The parasitemia rose more rapidly than expected and the horse had to be euthanized after only 4 days of tick feeding (10 days post inoculation). Ticks were fed on the acute horse separated by species and sex. After removal from the acquisition host they were incubated, 7 days for *A. cajennense*, and 11 days for *D. variabilis*, then each group was applied to a separate transmission host. Males were allowed to reattach and feed for 7 days, females were placed on the host with an equal number of uninfected males to stimulate reattachment and feeding, and were allowed to feed to repletion. Transmission fed males, and a sample of transmission fed females, were dissected and their guts and salivary glands tested by PCR for *T. equi* (Table 5).

**Table 5:** **Intrastadial transmission Experiment 1, adult ticks were acquisition fed on splenectomized horse Ho-226 for 4 days; parasitemia averaged 6.21 × 10**^**7 **^**genome copies per ml of blood over the acquisition feeding period, as determined by qPCR**

**Horse #**	**Tick species**	**Sex**	**Incubation (days)**^***a***^	**Transmission feed (days)**	**# recovered/# applied**	**# SG pos/# tested**	**# pos eggs/# ovip**^***b***^
Ho-220	*A. caj*	♂♂	7	7	47/76	0/46	
Ho-216		♀♀	7	7-10^*c*^	33/78	0/7	0/26
Ho-214	*D. var*	♂♂	11	7	98/100	0/98	
Ho-219		♀♀	11	6-9^*c*^	97/100	0/11	0/86

After acquisition feeding ticks were placed on 4 different hosts for transmission feeding. None of the transmission hosts acquired infection. After transmission feeding all males and a sample of females were dissected and their salivary glands tested for infection by nPCR. All remaining females that had fed to repletion were held for oviposition and their eggs tested for presence of *T. equi* DNA.

^*a*^Time between acquisition feed and transmission feed.

^*b*^Number of ticks laying positive egg masses/ total number of egg masses tested.

^*c*^Reflects the amount of time required for female ticks to feed to repletion and drop off.

### Intrastadial transmission, experiment 2

A second Intrastadial transmission experiment was performed on spleen intact horse Ho-266 to allow longer acquisition feedings, a longer incubation period between acquisition and transmission, and longer transmission feeding times for both *A. cajennense*, and *D. variabilis*. Males and females were fed in separate patches to prolong feeding time (in the absence of males most unmated females will not feed to repletion, but remain attached until manually removed). Males were acquisition fed for 14 days; unmated, partially fed females of both species were removed after 11 days of acquisition feeding. Acquisition fed ticks were incubated, males for 16 days, females for 19 days, then allowed to reattach and feed. *A. cajennense* males and females were put in separate patches on Ho-209, *D. variabilis* males and females were likewise put on Ho-211. A sample of acquisition fed males and females was dissected prior to transmission feeding, and another sample dissected after transmission, and their guts and salivary glands tested by PCR for *T. equi* (Table 6).

**Table 6:** **Intrastadial transmission Experiment 2, adult ticks were acquisition fed on *T. equi *** **infected horse Ho-266; males for 14 days (parasitemia averaged 1.19 × 10**^**5 **^**genome copies per ml of blood over the 11 day acquisition feeding period, as determined by qPCR), females for 11 days (9.67 × 10**^**4**^**)**

**Horse #**	**Tick species**	**Sex**	**Incubation (days)**^***a***^	**Transmission feed (days)**	**# recovered/# applied**	**# gut pos/# tested**	**# SG pos/# tested**
Ho-209	*A. caj*	♂♂	16	18	2/69	0/2	0/2
		♀♀^*b*^	19	9-18^*c*^	8/51	2/3	0/3
Ho-211	*D. var*	♂♂	16	18	15/33	0/15	0/15
		♀♀^*b*^	19	6-18^*c*^	60/66	0/15	0/15

After acquisition feeding *A. cajennense* were placed on Ho-209 and *D. variabilis* on Ho- 211 for transmission feeding. Ho-209 acquired infection. After transmission feeding all males and a sample of females were dissected and their guts and salivary glands tested for infection by nPCR.

^*a*^Time between acquisition feed and transmission feed.

^*b*^Partially engorged females put on for reattachment.

^*c*^Reflects the amount of time required for female ticks to feed to repletion and drop off.

### Transovarial passage

To determine if there is the potential for transovarial passage both *D. variabilis* and *A. cajennense* females that were partially fed during acute infection on Ho-266 (see above) and then fed to repletion on uninfected horses, were held for oviposition. DNA was isolated from a sample of ≈ 100 eggs from the egg mass of each individual female and tested for the presence of *T. equi* DNA by nPCR. Larval transmission feeding was not attempted.

## Results

### Transstadial transmission of *T. equi*

Transmission did not occur for either tick species in either of the transstadial transmission experiments. During the acquisition feeding for the first transstadial transmission trial acquisition hosts Ho-183 and Ho-198 had levels of infection of 1.4 × 10^4^ (4.15 Logs) and 2.1 × 10^5^ (5.32 Logs) copies per ml of blood respectively, as determined by qPCR (Table 1). Molting of the acquisition fed *A. cajennense* nymphs was complete 14–21 days after repletion and the freshly molted adults were transmission fed on horses Ho-259 and Ho-261 about 14 days later, as soon as they were ready to feed (Table 2). The *D. variabilis* nymphs took less time to molt (≈7–14 days) but were not fed until approximately 100 days later, when they were placed on Ho-138 for transmission feeding (Table 3). Ho-259, Ho-261 and Ho-138 were monitored for infection as described and no infection was detected. None of the dissected ticks of either species had PCR positive salivary glands (Tables 1–3).

In the second transstadial transmission trial the average infection level of the acquisition horse, Ho-266, over the 6 days during which the majority of the *A. cajennense* nymphs completed their acquisition feeding was 1.16 × 10^5^ (5.06 Logs) copies per ml of blood. Engorged nymphs were tested for *T. equi* by nPCR immediately after repletion and all (n = 19) were below detection. The remaining replete nymphs molted to adults in 14–21 days and were transmission fed on Ho-212 approximately 50 days later. The transmission horse, Ho-212, was monitored for infection, as described, no infection was detected. None of the transmission fed adult ticks were nPCR positive either before or after molting or after transmission feeding (Table 4).

### Intrastadial transmission of *T. equi*

*Amblyomma cajennense* transmitted *T. equi* intrastadially only when very long acquisition and transmission feedings were used, but failed to transmit with shorter feeding times. *Dermacentor variabilis* did not transmit by this route with either short or longer feeding intervals. In the first trial the level of *T. equi* infection in the acquisition horse, Ho-226, during the 4 days of the acquisition feeding, as determined by qPCR, was 6.21 × 10^7^ (7.79 Logs) copies per ml of blood. *A. cajennense* were held for 7 days, and *D. variabilis* for 11 days before being applied to horses for a transmission feeding. Male ticks of both species were transmission fed for 7 days, females were fed to repletion (stimulated by the presence of uninfected males) and took 7–10 days for *A. cajennense* and 6–9 days for *D. variabilis*. The transmission horses, Ho-220, Ho-216, Ho-214 and Ho-219, were monitored, as described, and did not acquire infections. No *T. equi* infection was detected in any of the tick tissues after transmission feeding (Table 5).

In the second intrastadial transmission trial longer feeding and incubation times were used. The acquisition host was Ho-266, the same animal used for the second transstadial transmission study described above; qPCR results for this animal are reported above for the first 6 days of feeding, however, the adults were left on to continue feeding after the replete nymphs dropped off. Total acquisition feeding time for the females of both species was 11 days and the average parasitemia over this time was 9.67 × 10^4^ (4.99 Logs) copies per ml of blood. Acquisition feeding time for the males was 14 days and the average parasitemia over this time was 1.19 × 10^5^ (5.08 Logs) copies per ml of blood. After acquisition feeding ticks were incubated at 26˚C; males for 16 and females for 19 days prior to transmission feeding. A sample of acquisition fed ticks was dissected immediately after feeding and another sample after 8 days of incubation. For *A. cajennense* 40% of males (4/10) and females (4/10) had nPCR positive guts immediately after acquisition, by 8 days guts were negative (0/9) but 22% (2/9) of the ticks had PCR positive salivary glands. For *D. variabilis* fewer ticks were available for dissection and only freshly fed females were dissected; 20% (2/10) had nPCR positive guts. The remaining ticks were transmission fed; *A. cajennense* were transmission fed on horse Ho-209, males for 18 days, females until they dropped off at repletion (9–18 days). This horse was PCR positive 18 days after tick application. The *D. variabilis* were transmission fed on horse, Ho-211, in a similar fashion with the exception that the first replete females began dropping off at 6 days. Transmission did not occur, this horse remained negative. A sample of acquisition fed males and females was dissected before and after transmission feeding and their gut and salivary glands tested by PCR for *T. equi* (Table 6). After an 11 day acquisition feed a small portion of females of either species had nPCR positive guts. After the 14 day acquisition feed a small portion of the *A. cajennense* had nPCR positive guts and salivary glands, *D. variabilis* males were not tested after acquisition feeding. After transmission feeding none of the *D. variabilis* had nPCR positive guts or salivary glands, whereas 2 of the 3 *A. cajennense* that remained alive at the end of the feeding had positive guts, their salivary glands were negative.

### Transovarial passage of *T. equi*

A sample of the eggs laid by each of the 86 *D. variabilis* and 26 *A. cajennense* females that had acquisition fed on Ho-226 (see above for level of infection) were tested for the presence of *T. equi* DNA by nPCR. All egg samples were negative and consequently transmission feeding was not attempted.

## Discussion

These studies with laboratory reared ticks confirm that *A. cajennense* is a competent intrastadial vector of *T. equi*, validating the earlier observation by Scoles *et al.* made from field collected ticks [7]. Intrastadial transmission failed when the ticks had a short acquisition feed on a highly parasitemic host, coupled with a short incubation period and a relatively short transmission feed (for a total time from the beginning of acquisition to the end of transmission of 18–21 days for male and female ticks respectively). However, intrastadial transmission was successful when more time was available for development of the parasite from uptake by the vector to the point when the tick was able to transmit (i.e. the extrinsic incubation period, or EIP). In this case the total time between the beginning of the acquisition feed to the end of the transmission feeding was 48 days, Transmission was successful during this longer EIP even though the parasitemia of the acquisition host was more than 2 orders of magnitude lower (4.98-5.08 vs. 7.79 logs). These results suggest that the length of the EIP may play a more critical role in transmission than parasitemia. The presence of the parasite in the salivary glands of ticks dissected after acquisition feeding confirms that this is biological, not mechanical transmission.

It is important to note that under natural field conditions once a male tick has acquired a host it would be unlikely to be out of contact with that or a subsequent host for any time other than the very short interval required for inter-host movement. The off host incubation times used in these studies are a necessary component of the experimental design to ensure that there is no mechanical transmission. Although the off-host incubations used in these experiments are artificial and their duration is arbitrarily chosen, they are carried out at 26˚C and consequently they are not dissimilar to the time the tick might spend moving around on the host seeking mating opportunities, but not blood feeding.

Transstadial transmission (nymphal acquisition, adult transmission) failed for *A. cajennense* in both trials, confirming the observation of Ribeiro and co-workers (2011) and suggesting that this species is not a competent transstadial vector [14]. For these studies we tested two different transstadial EIPs, 14 days from molt to transmission feed in the first experiment and 50 days in the second. In both of these experiments the parasitemia of the acquisition host was similar to the parasitemia from which intrastadial transmission was successful, however, even at levels of parasitemia that were sufficient for intrastadial transmission, transstadial transmission did not occur. This may be due to the very small amount of blood ingested by nymphs as compared to the relatively larger amount that would be taken by an adult tick, it is possible that nymphal ticks feeding on an acutely infected splenectomized host with a very high parasitemia would be competent to transmit, but this experiment is yet to be carried out.

Although transovarial transmission was not tested directly by transmission feeding larval offspring from females fed on an infected horse, negative nPCR on the eggs laid by these females suggests that transovarial passage did not occur. Previous authors have detected *T. equi* DNA in eggs and larvae of *Dermacentor nuttalli* that had been fed on *T. equi* infected horses but transmission was not attempted [18]. Transmission did not occur in similar studies with *B. microplus* carried through to transmission feeding, even though the eggs were PCR positive and came from females that were hemolymph positive for *T. equi*[4]. Although the data we present here is not definitive, it appears that transovarial transmission of *T. equi* by *A. cajennense* or *D. variabilis* is unlikely, or at least inefficient. Other evidence from our laboratory (data not shown) also supports this conclusion; the tick colonies used in these studies were established from ticks collected from seropositive horses in the field, and although the field collected adult ticks transmitted to naive horses under laboratory conditions [7], the larval offspring were confirmed to be free of infection when fed on naive horses for colonization.

*D. variabilis* failed to transmit *T. equi*, either transstadially or intrastadially in these investigations, even though this species has been shown to be a competent intrastadial vector in previously published studies [7,11]. Since the tick colony used for these studies was established from the same field collected adult ticks that transmitted *T. equi* to the horse that served as the source of the isolate used in these studies [7], we know that failure of transmission was not due to a lack of vector competence. Instead these data may suggest that *D. variabilis* has a low vector capacity (i.e. it is an inefficient vector for *T. equi*). Since we already know it is a competent vector, failure of transmission in this case may simply suggest that our study design did not provide the conditions necessary for efficient transmission. For example it may be possible that using a greater number of ticks, a higher parasitemia, a longer extrinsic incubation period, or some combination of these factors would have resulted in transmission. The work presented in this paper represents the first steps towards development of a reliable and repeatable splenectomized horse acquisition and transmission model for testing a wider cross section of suspected vector species.

*Amblyomma cajennense* is commonly reported from horses in South America and in spite of it being implicated as a vector through epidemiological observations [12,13] it had not previously been shown to be transmission competent until the report of Scoles *et al.* (2011) in which field collected ticks from the Texas *T. equi* outbreak were transferred from infected to uninfected horses and allowed to reattach and resume feeding (intrastadial transmission) [7]. During the investigation of the Texas outbreak *A. cajennense* was found on 79% of the horses, and the high prevalence of *T. equi* infection (reaching 100% in some ranch divisions) suggested a focus of tick-borne transmission. Prior to the Texas outbreak the only experimental vectors of *T. equi* found in the United States were *R. microplus* and *D. variabilis*[10,11]. Because *R. microplus* is a one-host tick and transovarial transmission has not been shown to occur in this species, transmission by *R. microplus* would require movement of the ticks between hosts. Cattle are the primary hosts for *R. microplus*, and it is rarely found on horses unless they are pastured along with cattle [19], so even though it is vector competent, epidemiological considerations suggest that *R. microplus* is unlikely to be a very efficient natural vector for *T. equi*. Furthermore, the Texas outbreak ranch is well north of the quarantine zone in an area that is reportedly free of *R. microplus*. So, although it is possible that an outbreak population of *R. microplus* has occurred in the outbreak area, no ticks of this species were found on horses at the time of the outbreak investigation [7]. Furthermore, this species has not been reported to be present on cattle at the outbreak ranch for many years.

Although intrastadial transmission would be necessary for a one-host tick like *R. microplus* to transmit *T. equi* (in the absence of transovarial transmission), interstadial transmission (i.e. acquisition by one feeding stage of the tick and subsequently transmission by the following tick stage after molting) might be expected to be the most efficient means of transmission for a three-host tick such as *A. cajennense*. In light of the fact that interstadial transmission has not been demonstrated in this or previous studies [14], failure to experimentally implicate *A. cajennense* as a vector by anything other than epidemiological inference [13] may be due to the failure to recognize intrastadial transmission as an epidemiologically meaningful and potentially important means of transmission. This is in spite of the fact that intrastadial transmission has previously been demonstrated experimentally for this parasite in at least two different tick species occurring in the U.S.: *D. variabilis* and *R. microplus*[11,16], and may have been described as early as 1955 for transmission of *T. equi* by *Hyalomma marginatum* and *Dermacentor marginatus* by Russian researchers working independently from one another [20,21]. It has long been understood that intrastadial transmission is the primary means of transmission for the bacterial pathogen *A. marginale* in the areas of the U.S. where its primary vectors are *Dermacentor* ticks [22-24], but intrastadial transmission of an apicomplexan parasite may represent something of a paradigm shift.

For biological intrastadial transmission to occur all of the steps in the parasite life cycle must be occurring within the adult stage, including: formation of haploid sexual stages in the gut; fusion of these gametes and formation of the diploid kinete; movement of the kinete to and invasion of the salivary glands where haploid sporozoites are formed and subsequently transmitted in the saliva as the tick feeds. A relatively long extrinsic incubation period may be required for all of these steps to be completed before transmission. Male *A. cajennense* are reported to have feeding intervals as long as 86 days [25], this time frame of nearly 3 months should be more than sufficient for parasite development.

Although intrastadial transmission seems counter intuitive on first examination because it requires tick movement between hosts, movement of male Metastriate ticks between hosts is consistent with their reproductive biology. After a short period of blood feeding, which is required for sperm maturation, male Metastriate ticks detach and move around, seeking females with which to mate [26]. Movement of male ticks between hosts has previously been demonstrated [27,28]. Furthermore, in the case of *A. cajennense* and *T. equi*, intrastadial transmission may increase the efficiency of transmission over that of transstadial transmission by increasing the probability of acquisition fed ticks encountering a susceptible transmission host. Immature *A. cajennense* have a very broad host range [29] so they are less likely than adults would be to acquisition feed on an infected equine. The host range of adults is much narrower and they are commonly associated with horses, consequently, an adult is more likely to encounter an equine host, and once it has, the gregarious nature of equines [30] makes it more likely that interhost movement would be to another susceptible host. Furthermore, because male *A. cajennense* were reported to remain fertile for the full 86 days of their feeding interval [25] they would presumably continue to move around seeking females to mate during this time. This long period of survival and activity provides a very broad window for parasite development, as well as for movement between hosts, and consequently for biological intrastadial transmission.

## Conclusions

*Amblyomma cajennense* is an intrastadial vector of *T. equi.* This species has long been suspected as a vector in South America based on epidemiological data but actual experimental demonstration of vector competence has not been forthcoming. Previous vector competence studies have emphasized transstadial transmission but it now appears that intrastadial transmission may be epidemiologically meaningful. Intrastadial biological transmission has been confirmed for *A. cajennense* in this study and we hypothesize that movement of male ticks between hosts as they seek females for mating may be the natural mode of transmission of *T. equi* by this species. We also suggest that a long extrinsic incubation period is required in order to allow the parasite to complete its life cycle within the tick, and that the long survival times of male *A. cajennense* may facilitate transmission by allowing time for this development. Furthermore, considering the gregarious nature of horses, intrastadial transmission may also be a mechanism for increasing the likelihood of transmission by increasing the probability that the tick will encounter a second susceptible host of the same species for transmission. As we work towards implication of additional vector species it will be important to consider intrastadial transmission as one equally possible transmission scenario.

## Competing interests

The authors declare that they have no competing interests.

## Authors’ contributions

GAS conceived, designed and carried out these experiments (with technical assistance from those listed in the acknowledgements), and drafted the manuscript. MWU provided input on experimental design, provided hands on assistance in conducting the experiments, and assisted in writing the manuscript. Both authors approved the final manuscript.

## Authors’ information

GAS is a Research Entomologist and MWU is a Research Veterinary Medical Officer with the United States Department of Agriculture, Agricultural Research Service, Animal Disease Research Unit located in the Department of Veterinary Microbiology and Pathology at the College of Veterinary Medicine, Washington State University, Pullman Washington, USA. The focus of their work is to conduct basic and applied research on tick-borne pathogens which cause persistent infectious disease in livestock and have important economic and trade implications for U.S. animal agriculture.
